# Ensino híbrido na formação em saúde: uma revisão sistemática**

**DOI:** 10.15649/cuidarte.2051

**Published:** 2022-08-08

**Authors:** Ana Carolina Bezerra de Lima Danielle, Christine Moura dos Santos, Sabrina Lima de Almeida, Ellen Lucena da Silva, Emanuela Batista Ferreira e Pereira

**Affiliations:** 1 Universidade de Pernambuco (UPE). Faculdade de Enfermagem Nossa Senhora das Graças (FENSG). Recife, Brasil. Email: carolina.lima@upe.br Universidade de Pernambuco Universidade de Pernambuco Faculdade de Enfermagem Nossa Senhora das Graças Recife Brazil carolina.lima@upe.br; 2 Professora Adjunta da FENSG/UPE. Membro Permanente do Programa Associado de Pós-Graduação em Enfermagem da Universidade de Pernambuco/Universidade Estadual da Paraíba (PAPGEnf-UPE/ UEPB). Recife, Brasil. Email: danielle.moura@upe.br Universidade Estadual da Paraíba Universidade Estadual da Paraíba Recife Brazil danielle.moura@upe.br; 3 Acadêmica de Enfermagem da Universidade de Pernambuco FENSG/UPE. Recife, Brasil. E-mail: sabrrinalima1999@gmail.com Universidade de Pernambuco Universidade de Pernambuco Recife Brazil sabrrinalima1999@gmail.com; 4 Acadêmica de Enfermagem da Universidade Estadual da Paraíba FENSG/UPE. Recife, Brasil. E-mail: ellenlucenaa@gmail.com Universidade Estadual da Paraíba Universidade Estadual da Paraíba Recife Brazil ellenlucenaa@gmail.com; 5 Professora Adjunta da Universidade Estadual da Paraíba FENSG/UPE. Recife, Brasil. E-mail: emanuela.pereira@upe.br Universidade Estadual da Paraíba Universidade Estadual da Paraíba Recife Brazil emanuela.pereira@upe.br

**Keywords:** Ensino, Educação à Distância, Educação superior, Aprendizagem, Revisão Sistemática, Teaching, Education, Distance, Education, Higher, Learning, Systematic Review, Enseñanza, Educación a Distancia, Educación Superior, Aprendizaje, Revisión sistemática

## Abstract

**Introdução:**

A partir da necessidade de reestruturação das instituições de ensino em saúde devido à pandemia da COVID-19, o ensino híbrido vem se destacando como possibilidade de reorganização das atividades educativas.

**O objetivo:**

deste estudo foi escrever o desenvolvimento do ensino híbrido na formação de profissionais da área da saúde.

**Materiais e Métodos:**

Revisão sis- temática da literatura, baseada nas recomendações da *Preferred Reporting Items for Systematic Reviews and Meta-Analyses* (PRISMA). A pesquisa foi desenvolvida em pares, entre julho a setembro de 2020, em quatro bases de dados eletrôni- cas. Os descritores foram os termos “Educação a Distância”, “Educação Superior”, “Aprendizagem”, “Saúde”, “Enfermagem”, “Medicina”, “Odontologia” e “Fisiote- rapia” e “Ensino híbrido”. Os artigos foram classificados conforme seu Nível de Evidência.

**Resultados:**

49 artigos foram selecionados, entre estudos quanti- tativos, qualitativos e de método misto. Foram encontradas experiências do desenvolvimento do ensino híbrido nos diferentes cursos de formação na área da saúde. Observou-se aplicação do ensino híbrido segundo o modelo de Ro- tação, modelo *à la carte* e o modelo Flex.

**Discussão:**

o ensino híbrido vem ganhando destaque cada vez maior no cenário da educação acadêmica em saúde. Foi visto que, a partir dele, o aluno destaca-se em sua aprendizagem, pois é o principal gerenciador deste processo, aprendendo ativamente por diversos ins- trumentos educativos a partir da condução do professor.

**Conclusões:**

O êxito do ensino híbrido pode estar relacionado ao seu caráter inovador, flexível, com boa relação custo-benefício e capaz de tornar os alunos protagonistas do seu processo de ensino-aprendizagem, influenciando no desempenho acadêmico dos alunos.

## Abstract (en)

**Introduction:**

Given the need to restructure healthcare education institutions due to the COVID-19 pandemic, hybrid education has been identified as an alternative to reorganize educational activities. The objective of this study is to describe hybrid education development in healthcare education.

**Materials and Methods:**

A systematic literature review was conducted based on the PRISMA (preferred reporting items for systematic reviews and meta-analyses) guideline. A peerreviewed search was conducted across four electronic databases between July and September 2020. Descriptors used were “distance education”, “learning”, “health”, “nursing”, “medicine”, “dentistry”, “physical therapy” and “hybrid education”. Selected articles were classified based on their level of evidence.

**Results:**

49 articles were selected, including quantitative, qualitative and mixed methods studies. Experiences in the development of hybrid education in multiple healthcare courses were found. Hybrid education has been implemented based on the rotation model, a la carte model and Flex model.

**Discussion:**

Hybrid education has been gaining more and more importance in the academic scenario of healthcare education. Based on this, students manage to stand out in their learning process as they become the main agent of this process by actively learning using multiple education tools provided by their teachers.

**Conclusions:**

Successful hybrid education can be related to its innovative, flexible, cost-effective nature and the ability to turn students into key players in their teaching-learning process, which ends up influencing their own academic performance.

## Resumen

**Introducción:**

Ante la necesidad de reestructurar las instituciones de educación en salud a causa de la pandemia por COVID-19, se ha señalado a la educación híbrida como una alternativa para reorganizar las actividades educativas. El objetivo de este estudio es describir el desarrollo de la educación híbrida en la formación de los profesionales en el área de la salud.

**Materiales y métodos:**

Revisión sistemática de la literatura basada en las recomendaciones de los elementos de informes preferidos para revisiones sistemáticas y metaanálisis (PRISMA). La búsqueda se realizó por pares entre julio y septiembre de 2020 en cuatro bases de datos electrónicas. Los descriptores utilizados fueron “educación a distancia”, “educación superior”, “aprendizaje”, “salud”, “enfermería”, “medicina”, “odontología”, “fisioterapia” y “educación híbrida”. A su vez, los artículos seleccionados se clasificaron según su nivel de evidencia.

**Resultados:**

Se seleccionaron 49 artículos, entre los que se incluyeron estudios cuantitativos, cualitativos y de métodos mixtos. Se identificaron experiencias en el desarrollo de la educación híbrida en diferentes cursos de formación en el área de la salud. Se observó que la educación híbrida se implementó de acuerdo con el modelo de rotación, el modelo a la carta y el modelo Flex.

**Discusión:**

La enseñanza híbrida ha venido ganando cada vez más importancia en el escenario académico de la educación en salud. Se observó que, a partir de esta, el estudiante logra destacarse en su aprendizaje, ya que se convierte en el principal gestor de este proceso al aprender de forma activa mediante diversas herramientas educativas guiadas por el docente.

**Conclusiones:**

El éxito de la educación híbrida se puede relacionar con su carácter innovador, flexible, con buena relación costo-beneficio y la habilidad de convertir a los estudiantes en protagonistas de su proceso de enseñanza-aprendizaje, lo que influye en su propio rendimiento académico.

## Introdução

A situação de pandemia causada pelo Novo Coronavírus (SARS-COV-2) foi responsável por provocar mudanças inesperadas em toda a sociedade, pois trouxe consigo a necessidade do isolamento social e de mudanças comportamentais com o intuito de diminuir a propagação do vírus^1^. Nesse contexto, as Instituições de Ensino Superior (IES) precisaram reestruturar seu modelo de ensino presencial e passaram a realizá-los de forma semipresencial ou totalmente à distância para atender às medidas sanitárias vigentes^2^,^3^.

A partir desta emergente necessidade de reestruturação, o ensino híbrido vem se destacando como uma possibilidade de organização do ensino nas instituições do ensino superior. Comumente chamado de aprendizagem combinada, aprendizado misto ou *“blended learning”*, tratase de um programa de educação formal no qual o aluno aprende em parte por meio do ensino *online*, com algum elemento de controle do estudante sobre o tempo, lugar, modo e/ou ritmo do estudo, e em parte em uma localidade física supervisionada, fora de sua residência^4^.

O ensino híbrido apresenta-se como uma estratégia educacional para os cursos da área da saúde no atual contexto da pandemia, onde a tecnologia poderá auxiliar na personalização da aprendizagem, assim como possibilitar ao aluno aprender no seu ritmo e de acordo com os seus conhecimentos prévios^5^. Há diferentes modelos de ensino híbrido, entre eles o modelo de Rotação, subdividido em Rotação por Estações de Trabalho, Laboratório Rotacional, Sala de Aula Invertida e Rotação Individual, e os modelos Flex, *À La Carte* e Virtual Enriquecido, onde todos eles permitem a associação da educação *online* à sala de aula presencial^6^.

Neste contexto, questiona-se: que tipos de estudos têm sido desenvolvidos a respeito do ensino híbrido, de onde se originam e a que nível e área da formação em saúde se destinam? Como esta abordagem tem sido implementada? Quais as melhores formas de combinar atividades presenciais e aquelas mediadas pelas Tecnologias Digitais de Informação e Comunicação (TDI- Cs)? Estes questionamentos tornam-se relevantes diante do cenário da inserção das TDICs na área da educação, incluindo o ensino em saúde, bem como no desenvolvimento das competências do século XXI.

De igual maneira, percebe-se a importância das TDICs no contexto da pandemia da COVID-19, pois existe uma busca pela compreensão das contribuições dessas ferramentas no processo de planejamento e retomada das atividades educacionais seguindo os protocolos da Organização Mundial da Saúde (OMS)^3^,^5^. Portanto, o objetivo deste estudo foi descrever o desenvolvimento do ensino híbrido na formação de profissionais da área da saúde.

## Materiais e Métodos

Trata-se de uma revisão sistemática da literatura, cuja questão de pesquisa foi estruturada conforme o acrônimo PICO^7^, onde a população foi composta pelos estudantes da área de saúde, a intervenção foi a utilização do ensino híbrido na formação em saúde, o controle foi o ensino tradicional e o desfecho foi a contribuição do ensino híbrido no processo de aprendizagem. A partir destas definições, foi formulada a seguinte questão norteadora: “Como vem sendo desenvolvido o ensino híbrido em instituições de ensino superior na área da saúde? ”

Foram adotados os seguintes critérios de elegibilidade: (a) artigos completos publicados em revistas científicas; (b) período de publicação de 2015 a 2020, devido à atualidade do tema e à busca pelos métodos e tecnologias mais recentes utilizadas no ensino híbrido em saúde; (c) idiomas Português, Inglês ou Espanhol; (d) artigos que respondessem à pergunta norteadora. Também foi definido o seguinte critério de exclusão: (a) artigos do tipo editorial, revisão de literatura, artigos de reflexão, teses e dissertações.

O processo de busca ocorreu em quatro bases eletrônicas: a *Medical Literature Analysis and Retrieval System Online* (MEDLINE/PubMed), a *Scientific Electronic Library Online* (SciElo), a Literatura Latino-Americana e do Caribe em Ciências da Saúde (LILACS) e Banco de Dados de Enfermagem (BDENF). Utilizou-se os descritores selecionados a partir do MeSH: *“Active learning”*, *“Health education”*, *“Medical education”*, *“Nursing education”*, *“Dentistry”* e *“Physical therapists”* e o termo derivado *“Blended learning”*.

No Descritores em Ciências da Saúde (DeCS), foram selecionados os termos “Educação a Distância”, “Educação Superior”, “Aprendizagem”, “Saúde”, “Enfermagem”, “Medicina”, “Odontologia” e “Fisioterapia” e o termo derivado “Ensino híbrido”. Para garantir a combinação entre os termos, utilizou-se o operador booleano AND.

A partir destes descritores, foram realizadas as seguintes combinações nas bases de dados: (a) PubMed: (blended learning) and (health education); (blended learning) and (medical education[mesh terms]); (blended learning) and (nursing education[mesh terms]); (blended learning) and (dentistry[mesh terms]); (blended learning) and (physical therapists[mesh terms]); (b) SciELO: (educação a distância) and (educação superior); (aprendizagem) and (educação a distância); (ensino híbrido); (ensino híbrido) and (saúde); (blended learning) and (saúde); (ensinohíbrido) and (enfermagem); (ensino híbrido) and (medicina); (ensino híbrido) and (odontologia); (ensinohíbrido) and (fisioterapia); (c) LILACS: (blended learning) and (health education); (ensinohíbrido) and (saúde); (d) BDENF: (ensino híbrido) and (saúde); ensino híbrido; educação a distância and educação superior.

**Figura 1:**
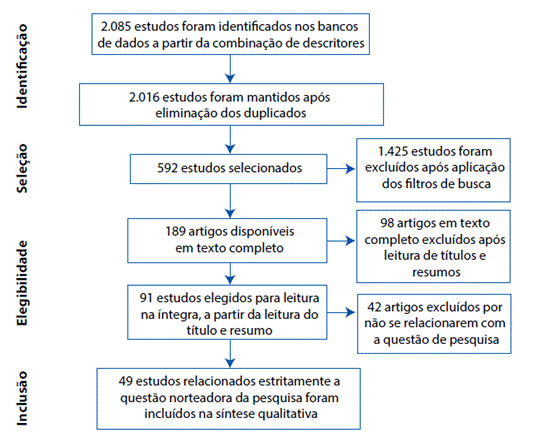
Fluxograma do processo de seleção dos estudos conforme a recomendação PRISMA.

A pesquisa eletrônica foi desenvolvida em pares, de forma independente e com base no mesmo protocolo de coleta, no período de 07 de julho a 19 de setembro de 2020. A partir das combinações supracitadas, deu-se início o processo de seleção dos estudos, conforme as orientações teóricas da *Preferred Reporting Items for Systematic Reviews and Meta-Analyses* (PRISMA)^8^, apresentado na Figura 1, nos resultados deste estudo.

Os dados dos artigos foram organizados em uma planilha padrão do Google Sheets® para serem analisados a partir dos seguintes elementos: base de dados em que o artigo foi encontrado, identificação do artigo (título, autores, ano, cidade e país), objetivo da pesquisa, desenho e métodos, tamanho da amostra, principais resultados e conclusões, tema central, nível em que o ensino híbrido foi aplicado e o modelo de ensino híbrido adotado. O banco de dados da pesquisa está armazenado no repositório *Mendeley Data*^9^.

Para a definição do Nível de Evidência dos estudos incluídos, atribuiu-se, de acordo com o seu delineamento: I para revisões sistemáticas e metanálise de ensaios clínicos randomizados; II para ensaios clínicos randomizados; III para ensaio controlado não randomizado; IV para estudos caso-controle ou coorte; V para revisões sistemáticas de estudos qualitativos ou descritivos; VI para estudos qualitativos ou descritivos; e VII para parecer de autoridades e/ou relatórios de comitês de especialistas. Esta hierarquia classifica os níveis I e II como fortes, III a V como moderados e VI a VII como fracos^10^.

Os artigos lidos na íntegra foram avaliados considerando os critérios: atinge o objetivo do estudo; apresenta a amostra da população compatível com o estudo; apresenta clareza na metodologia aplicada; apresenta resultados claros; descrição do artigo é compatível com o objeto da pesquisa. Assim, os artigos selecionados para análise resultaram da aplicação destes critérios e da leitura criteriosa, bem como a avaliação da qualidade e relevância para o estudo em questão.

## Resultados

Foram selecionadas 49 publicações, cujo diagrama de fluxo que detalha este processo encontrase ilustrado na Figura 1. Dentre as bases de dados escolhidas, 29 artigos (59,2%) encontravam-se na PubMed, 19 (38,8%) na LILACS e 01 (2,0%) na SciELO. A maior parte dos artigos foi publicada nos anos de 2019 e 2017, ambos com 10 artigos (20,4%), seguida dos anos de 2018 e 2016, ambos com 9 artigos (18,4%). Segundo o Nível de Evidência, foram encontrados 31 artigos com nível VI - Fraco (63,3%), 13 artigos com nível III a IV - Moderado (26,5%) e 05 artigos com nível II - Forte (10,2%).

Fonte: Adaptado de Moher et al., 2009^10^

Estas publicações envolveram amostras que, somadas, abrangeram 6.120 estudantes e professores investigados. Os estudos incluídos envolveram 39 estudos quantitativos (79,6%), 07 qualitativos (14,3%) e 03 estudos de método misto (6,1%). Quanto aos estudos quantitativos, a maioria utilizou delineamento transversal 17 (43,6%), seguidos do prospectivo 07 (17,9%) e dos ensaios clínicos controlados randomizados e estudos quase experimentais, cada um destes com 05 estudos (12,8%). As características gerais das publicações incluídas neste estudo encontramse detalhadas na Tabela 1.

**Tabela 1:** Síntese dos estudos incluídos na revisão sistemática sobre o ensino híbrido na formação em saúde. Recife - PE, 2020.

Autor (ano). Cidade, País. Código do estudo	Objetivos	Desenho	Amostra
Wu et al. (2020)^11^. Novena, Singapura. E1	Desenvolver e avaliar um programa de ensino híbrido de aprendizagem clínica para preceptores de enfermagem com o auxílio da pedagogia clínica baseada na web e aprendizagem baseada em casos.	Estudo prospectivo de grupo único	Enfermeiros preceptores de estudantes de graduaçao (n=150)
Sáiz-Manzanares et al. (2020)^12^. Burgos, Espanha. E2	Analisar dados de atividades online e presenciais dos alunos em um curso semipresencial de enfermagem.	Estudo experimental	Estudantes de graduação em enfermagem (n=120)
Halasa et al. (2020)^13^. Amman, Jordania. E3	Investigar a eficácia da aprendizagem combinada com um design de sala de aula invertida no desempenho acadêmico dos alunos de um curso de Bacharelado em Enfermagem.	Estudo quase experimental	Estudantes de graduação em enfermagem (n=125)
Fernandes et al. (2020)^14^. Recife, Brasil. E4	Planejar, desenvolver e avaliar um curso de especialização em gestão em oncologia por meio da utilização da metodologia do blended learning.	Estudo de coorte observacional	Estudantes de pós graduação de diversos cursos de saúde (n=45)
Lozano-Lozano et al. (2020)^15^. Granada, Espanha. E5	Examinar os efeitos de um método de aprendizagem combinada usando materiais tradicionais e um aplicativo de aprendizagem móvel (iPOT) entre alunos de graduação matriculados em um curso de ciência da saúde.	Ensaio clínico controlado randomizado	Estudantes de graduação em ciências da saúde (n=99)
Weber-Main (2019)^16^. Twin Cities, Estados Unidos. E6	Avaliar uma abordagem híbrida de treinamento de mentor, individualizado seguido por workshops baseados no currículo de Mentoreamento de Entrada.	Ensaio clínico controlado randomizado piloto	Professores universitários da área de saúde (n=59)
Shimizu et al. (2019)^17^. Matsumoto, Japão. E7	Investigar os efeitos da aprendizagem baseada em problemas (PBL) em associação com o blended learning (bPBL) em um grupo de estudantes de medicina asiáticos e o nível de aceitação dos elementos de e-learning.	Estudo prospectivo comparativo	Estudantes de graduação do quarto ano em medicina (n=96)
Røe et al. (2019)^18^. Oslo, Noruega. E8	Descrever o projeto, implementação e avaliação de uma abordagem de ensino em sala de aula invertida no ensino de fisioterapia.	Estudo prospectivo controlado	Estudantes de graduação em fisioterapia (n=54)
Naeem e Khan, (2019)^19^. Faisalabad, Parquistão. E9	Explorar os problemas enfrentados pelos alunos e facilidadores do programa híbrido do Mestrado em Educação de Profissionais da Saúde (MHPE) no Paquistão.	Estudo exploratório qualitativo	Estudantes e facilidadores do mestrado em saúde (n = 22)
Westerlaken et al. (2019)^20^. Utrecht, Holanda. E10	Descrever a introdução e avaliação de um novo conceito de educação para profissionais de saúde pós-graduados, desenvolvido com a abordagem de aprendizado misto, e discutir sobre o valor agregado da interação social.	Estudo qualitativo	Profissionais de saúde integrantes do mestrado (n=21
Moon e Hyun. (2019)^21^. Seoul, Coreia do Sul. E11	Examinar a eficácia de um programa de e-learning de reanimação cardiopulmonar (RCP) padronizado, desenvolvido por uma instituição de saúde pública coreana em colaboração com especialistas.	Ensaio clínico controlado randomizado.	Estudantes de graduação em enfermagem (n=120)
Engel et al. (2019)^22^. Bonn, Alemanha. E12	Examinar a viabilidade da prática da medicina baseada em evidências (EBM) por alunos durante o treinamento em medicina geral como parte de um conceito de ensino de aprendizagem combinada.	Estudo analítico transversal	Estudantes de graduação em medicina (n=35)
Rudd et al. (2019)^23^. Washington, USA. E13	Descrever um curso de eLearning desenvolvido pelo *Centers for Disease Control and Prevention* (CDC) sobre Sistemas de Informação em Saúde e os resultados do teste piloto na Namíbia e na Tanzânia.	Estudo observacional descritivo	Profissionais de saúde de diversas áreas (n=131)
Burrola-Mendez et al. (2019)^24^. Pensilvania, EUA. E14	Comparar a eficácia de ambas as metodologias de aprendizagem (cursos híbridos e presenciais) e a satisfação entre um grupo de fornecedores de serviços para cadeira de rodas na Índia e no México.	Estudo quase experimental controlado	Estudantes e profissionais de fisioterapia (n=81)
Yigzaw et al. (2019)^25^. Addis Ababa, Etiópia. E15	Avaliar uma abordagem de aprendizagem combinada para treinamento em emergência obstétrica e assistência neonatal.	Estudo quase experimental controlado	Profissionais de saúde de diversas áreas. (n=160)
Salim H, et al. (2018)^26^. Serdang, Malásia. E16	Explorar as percepções dos educadores e alunos de um Programa de Mestrado em Medicina de Família sobre a aprendizagem combinada adotada no programa.	Estudo qualitativo	Estudantes do mestrado em Medicina de Família (n = 17)
Furnes et al. (2018)^27^. Elverum, Noruega. E17	Explorar a avaliação dos alunos de graduação em enfermagem sobre os métodos de aprendizagem combinada para melhorar as habilidades de comunicação em enfermagem de saúde mental.	Estudo observacional descritivo	Estudantes degraduação em enfermagem (n=100)
Sheikhaboumasoudi et al. (2018)^28^. Isfahan, Iran. E18	Comparar o efeito da aprendizagem combinada (e-learning com os métodos tradicionais) com a aprendizagem tradicional nas pontuações e satisfação dos estudantes de enfermagem.	Estudo transversal comparativo	Estudantes de graduação de enfermagem (n=119)
McCutcheon et al. (2018)^29^. Belfast, Irlanda do Norte. E19	Testar se os alunos de graduação em enfermagem que receberam treinamento de habilidades de supervisores clínicos por meio de uma abordagem de aprendizagem combinada teriam pontuação mais alta em relação à supervisão clínica.	Ensaio clínico controlado randomizado	Estudantes de graduação de enfermagem (n=122)
Könings et al. (2018)^30^. Maastricht, Holanda. E20	Explorar as percepções dos alunos sobre a eficácia de um curso de liderança em saúde pública usando métodos de aprendizagem combinada e baseada em problemas (PBL).	Estudo transversal de avaliação	Profissionais de saúde (n= 19)
Balasubramaniam et al. (2018)^31^. Patna, Índia. E21	Avaliar a eficácia do modelo de abordagem de aprendizagem combinada em um programa de treinamento de enfermeiras auxiliares obstétricas.	Estudo transversal comparativo	Estudantes de graduação de enfermagem (n=136)
Micheal et al. (2018)^32^. Nova Gales do Sul, Austrália. E22	Descrever as lições aprendidas com uma inovação para ensinar gênero e sexualidade na Western Sydney University School of Medicine.	Estudo transversal de avaliação	Estudantes de graduação em medicina (n= 121)
Aguilar-Rodrígues et al. (2018)^33^. Valência, Espanha. E23	Avaliar o efeito de um modelo de blended learning na atitude, conhecimentos e opiniões dos estudantes de fisioterapia para a aprendizagem da ética profissional.	Ensaio clínico simples	Estudantes de graduação em fisioterapia (n=129)
Wilbur et al. (2018)^34^. Bath, Reino Unido. E24	Explorar as experiências de farmacêuticos do Oriente Médio matriculados em cursos de prática farmacêutica avançada ministrados por em ambiente de aprendizagem combinada.	Estudo qualitativo	Estudantes de pós-graduação em farmácia (n= 17)
Anderson e Krichbaum. (2017)^35^. Minneapolis, EUA. E25	Comparar os resultados de aprendizagem dos alunos e comparar a satisfação do aluno com o instrutor entre os alunos matriculados em duas sessões híbridas de um curso de fisiologia.	Estudo quase experimental comparativo	Estudantes de graduação de diversas áreas da saúde (n=235)
Herbert et al. (2017)^36^. Sydney, Austrália. E26	Avaliar a adoção de uma abordagem de aprendizado misto para grupos grandes, bem como a percepção do aluno sobre o papel dos recursos on-line para seu aprendizado.	Estudo transversal de avaliação	Estudantes de graduação em medicina (n= 264)
Luo et al. (2017)^37^. Dingjiaqiao, China. E27	Determinar se as características específicas dos alunos estavam relacionadas aos conhecimentos, atitudes e práticas do e-learning (CAP).	Estudo transversal	Estudantes de graduação em medicina (n= 119)
Tirmizi et al. (2017)^38^. Ontário, Canadá. E28	Apresentar os resultados de um estudo quase experimental realizado em 4 distritos em Badakshan, para melhoria do conhecimento entre profissionais de saúde sobre depressão.	Estudo quase experimental	Profissionais de saúde de diversas áreas (n=93)
Fermozelli et al. (2017)^39^. São Paulo, Brasil. E29	Avaliar, sob a ótica de estudantes de medicina, a motivação e a capacidade de contextualização proporcionada pela associação do blended learning ao ensino de patologia geral.	Estudo exploratório quantitativo	Estudantes de graduação em medicina (n=107)
Green et al. (2017)^40^. Victoria, Austrália. E30	Determinar a contribuição dos recursos de aprendizagem online para a aprendizagem do aluno em uma disciplina de anatomia combinada.	Estudo exploratório	Estudantes de graduação em fisioterapia (n=500)
Extavour et al. (2017)^41^. Champs Fleurs, Trindade e Tobago. E31	Avaliar as percepções de estudantes de farmácia sobre o ensino híbrido em um curso de seminário de farmácia.	Estudo transversal de avaliação	Estudantes de graduação em farmácia (n= 72)
Milic et al. (2017)^42^. Belgrado, Sérvia. E32	Determinar se a aprendizagem combinada é uma estratégia eficaz para adquirir competência em bioestatística em saúde pública.	Estudo prospectivo de coorte	Estudantes de mestrado em saúde pública (n=69)
Manyazewal et al. (2017)^43^. Addis Ababa, Etiópia. E33	Implementar e avaliar a eficácia de um curso com ensino híbrido sobre diagnóstico de tuberculose para profissionais de saúde da Etiópia.	Estudo transversal de avaliação	Profissionais de saúde de diversas áreas (n= 108)
Sonesson et al. (2017)^44^. Estolcomo, Suécia. E34	Analisar os desafios educacionais e investigar o uso potencial da aprendizagem combinada em cuidados avançados de trauma civil e militar.	Estudo qualitativo	Profissionais da área de medicina e enfermagem (n=51)
Morton et al. (2016)^45^. Londres, Reino Unido. E35	Avaliar as percepções de alunos de medicina sobre um módulo combinado, enfatizando os fatores que otimizam o envolvimento dos alunos e como eles perceberam a aprendizagem combinada.	Estudo de método misto	Estudantes de graduação em medicina (n=19)
Keleekai et al. (2016)^46^. Bethlehem, Estados Unidos. E36	Determinar o impacto de um programa instrucional abrangente e combinado sobre inserção de cateter venoso periférico (PIVC).	Ensaio clínico randomizado com desenho cruzado	Profissionais da área de enfermagem (n=36)
Milic et al. (2016)^47^. Belgrado, Sérvia. E37	Implementar o ensino híbrido ao ensino tradicional para avaliar o valor do aprendizado baseado na internet no ensino das estatísticas médicas; explorar a relação entre as características dos alunos e o resultado das estatísticas.	Estudo prospectivo	Estudantes de graduação em medicina (n=440)
Page et al. (2016)^48^. Victoria, Austrália. E38	Avaliar o impacto dos recursos de aprendizagem online nas percepções dos alunos sobre suas experiências de aprendizagem.	Estudo transversal comparativo	Estudantes de graduação em saúde de diversas áreas (n=609)
Sigaroudi et al. (2016)^49^. Teerã, Irã. E39	Compreender as experiências dos estudantes de doutorado em enfermagem relacionadas ao aprendizado misto.	Estudo qualitativo	Estudantes de doutorado em enfermagem (n=8)
Atkins et al. (2016)^50^. Estolcomo Suécia. E40	Avaliar a experiência do aluno em participar de cinco cursos “ARCADE BL” implementados de forma colaborativa em instituições da África, Ásia e Europa.	Estudo transversal avaliativo	Estudantes de graduação em saúde de diversas áreas (n=118)
Protsiv, et al. (2016)^51^. Estolcomo, Suécia. E41	Explorar os pontos de vista dos professores de cursos com ensino híbrido no contexto de dois projetos de capacitação de pesquisa colaborativa norte-sul.	Estudo qualitativo de avaliação	Professores da área da saúde (n=11)
Liebert, et al. (2016)^52^. Califórnia, Estados Unidos. E42	Investigar as percepções dos estudantes de medicina de uma sala de aula invertida para o estágio em cirurgia e sugerir as melhores práticas para implementação neste ambiente.	Estudo de método misto	Estudantes de graduação em medicina (n=89)
Te Pas et al. (2016)^53^. Amsterdã, Holanda. E43	Identificar os fatores que influenciam a percepção sobre a logística da aprendizagem combinada para alunos em Educação Médica em medicina baseada em evidências.	Estudo transversal	Preceptores de clínica médica da área de medicina (n=170)
Reissmann et al. (2015)^54^. Hamburgo, Alemanha. E44	Avaliar o uso do ensino híbrido em um curso pré-clínico em odontologia protética em uma escola de odontologia da Alemanha.	Estudo transversal de avaliação.	Estudantes de graduação em odontologia (n=71)
Belfi et al. (2015)^55^. Nova Iorque, USA. E45	Integrar a metodologia de aprendizagem combinada em um estágio introdutório "invertido" em radiologia e avaliar o impacto dessa abordagem na experiência educacional do aluno (desempenho e percepção).	Estudo transversal comparativo	Estudantes de graduação em medicina (n=101)
Patterson et al. (2015)^56^. Detroit, EUA. E46	Examinar a conclusão de um curso baseado em aprendizagem combinada para profissionais de saúde que tinham interesse em expandir sua carreira.	Estudo transversal	Estudantes de graduação em saúde de diversas áreas (n=308)
Hanson, et al. (2015)^57^. Queensland, Austrália. E47	Elucidar as perspectivas dos alunos sobre a turma invertida e determinar se essa abordagem poderia aumentar a compreensão que os alunos de graduação em enfermagem têm da ciência das drogas e sua aplicação na prática clínica.	Estudo transversal	Estudantes de graduação em enfermagem (n=407)
Castro, et al. (2015)^58^. Washington, USA. E48	Descrever como abordagens de aprendizagem online e em sala de aula foram usadas para projetar e oferecer um curso de enfermagem em saúde ocupacional em uma região multiestadual do noroeste dos Estados Unidos.	Estudo descritivo	Enfermeiros preceptores (não envolveu amostra de participantes)
Bosner, et al. (2015)^59^. Magburg, Alemanha. E49	Avaliar o impacto de um programa de aprendizagem combinada na satisfação dos alunos e no desenvolvimento de habilidades e conhecimentos.	Estudo de método misto	Estudantes de graduação em medicina (n=17)

Fonte: Elaboração própria.

Foram encontradas experiências do ensino híbrido no contexto de 18 cursos multiprofissionais (36,7%), 13 da área de medicina (26,5%) e 11 da área de enfermagem (22,4%). As publicações envolveram 30 estudos no nível de ensino da graduação (61,2%), 12 estudos realizados em cursos de capacitação para profissionais de saúde (24,5%) e 07 estudos no nível de pós-graduação (14,3%), sendo estes realizados com estudantes de mestrado (n=4, 57,1%), especialização (n=2, 28,6%) e doutorado (n=1, 14,3%).

Com relação ao modelo de ensino híbrido, 41 estudos compreenderam o modelo de Rotação (83,7%), 06 seguiram o modelo *À la carte* (12,2%) e 02 seguiram o modelo Flex (4,1%). Neste universo, importa destacar o modelo de Sala de Aula Invertida, que compreendeu um valor de 87,8% das intervenções guiadas pelo modelo de Rotação.

Com relação às estratégias de ensino-aprendizagem, identificou-se que foram utilizados nas atividades presenciais palestras ministradas por professores^13^,^21^,^24^,^26^,^36^,^39^,^50^,^54^,^55^,^59^ e oficinas e wo rkshops^11^,^16^,^21^,^24^,^36^,^38^,^40^,^48^, assim como os seminários^22^, leitura de artigos científicos e de material textual complementar^16^,^20^,^24^.

Além disso, foram realizadas atividades que envolveram metodologias ativas, entre elas a aprendizagem baseada em problemas^17^, aprendizagem baseada em projetos, sala de aula invertida^18^ e atividades culturais^14^. Estas atividades também contaram com prática nos laboratórios de habilidades e em clínicas anexas às instituições que ofertaram o ensino híbrido^14^,^31^,^36^,^40^,^46^,^56^,^59^.

Nas atividades *online*, os programas educacionais contaram com ambientes virtuais como o Moodle^11^,^36^,^37^,^38^,^41^,^49^ e outras plataformas tecnológicas e operacionais^13^,^14^,^36^,^37^,^38^,^44^,^54^. Nestes ambientes, predominaram os vídeos instrucionais^12^,^17^,^20^,^26^,^33^,^37^,^38^,^39^,^51^,^53^,^58^ e as palestras com especialistas^21^,^27^,^32^,^48^,^50^,^58^. Além destes, recursos hipermídia como textos, sons e animação^12^,^24^,^38^, fóruns de discussão^14^,^22^,^28^,^29^,^21^,^57^, portfólios, leitura de materiais didáticos, tutorias *online*, quizzes^14^,^22^,^28^,^36^,^38^,^39^,^42^,^43^,^57^ e casos clínicos^22^,^26^,^32^,^46^,^57^ também foram utilizados.

Houve adaptação de atividades tradicionalmente presenciais para ensino *online* por meio da simulação realística, cenários de caso e demonstrações ao vivo utilizando manequins para a gravação em vídeo^27^,^31^,^46^,^54^,^55^. Os alunos avaliaram positivamente os módulos *online* combinados com o estágio prático, especialmente a interatividade e a capacidade de retornar aos materiais em outros momentos no futuro^55^, tendo assim o controle do ritmo de sua aprendizagem, dando tempo para revisar conforme necessário^24^,^41^,^47^,^58^.

As atividades híbridas aumentaram a aprendizagem percebida e os níveis de satisfação dos estudantes, principalmente pela flexibilidade de horários e locais. As redes sociais foram recomendadasparaaumentaressainteração. Asinteraçõessociaisforamavaliadaspositivamente nas atividades online, o que contribuiu com um maior aproveitamento do ensino presencial. Como os alunos já tinham estudado previamente o assunto, eles contribuíram mais com as discussões presenciais^20^,^26^.

Os estudos analisados apontaram aspectos relacionados ao ganho significativo em habilidades e conhecimento^41^,^59^, ao maior envolvimento dos estudantes com o processo de aprendizagem e aumento do conhecimento autorrelatado dos alunos sobre questões relacionadas ao objeto de aprendizagem. Os baixos custos, facilidades para receber informações e um menor deslocamento também foram pontuados como benefícios deste modelo de ensino^25^.

Com relação às fragilidades e⁄ou desafios encontrados, foi relatado como negativa a quantidade extensa de atividades *online*^16^ motivação e autorregulação do estudante, inflexibilidade dos alunos em aceitar novas metodologias de aprendizagem e aprender de forma ativa, acomodação com o ensino tradicional, dificuldade em entender as instruções para a realização de tarefas, problemas tecnológicos de acesso a equipamentos e outras tecnologias^34^, falta de apoio dos facilitadores a respeito de disponibilidade e *feedback* oportuno, além de problemas na interação em grupo, falta de apoio estrutural^19^,^36^.

Em um dos estudos, as intervenções de natureza assíncrona, como o fórum de discussão *online* pode ter influenciado o baixo nível de captação, satisfação e experiência de aprendizado dos alunos com a modalidade *online*^29^. A carência de interação social presencial e a falta de alinhamento entre os conteúdos abordados de forma online e as palestras presenciais também foram destacados como desvantagens da aprendizagem combinada^36^.

## Discussão

O ensino híbrido é um método de ensino capaz de tornar o aluno protagonista do seu processo de aprendizagem, pois promove o seu envolvimento direto, participativo e reflexivo em todas as suas etapas, onde o compartilhamento de espaços, tempo, atividades, materiais, técnicas e tecnologias são componentes deste processo ativo^6^,^60^. Para os profissionais de saúde, foi evidenciado que esta abordagem híbrida permite desenvolver habilidades clínicas à medida que os estudantes podem revisar os materiais educativos no momento e local que escolherem, quantas vezes quiserem, aprimorando seu conhecimento e associando-o com as atividades práticas^20^.

Destaca-se, nesta revisão, que instituições educacionais de diversos países implementaram o ensino híbrido ou reformularam suas atividades em direção a ele pelas possibilidades de interação e flexibilidade que oferece aos estudantes e professores universitários. Em um dos estudos, foi percebido que, após a reestruturação do curso tradicional para o modelo combinado, o tempo mínimo necessário para concluir os módulos e atividades presenciais foi de 23,25h, em comparação às 26 h do formato tradicional, sendo o tempo restante utilizado para a apromoração de atividades práticas como treinamentos, simulações, discussão de casos clínicos e outras^36^.

Assim, é possível que o ensino híbrido se configure como uma abordagem relevante para o ensino universitário no cenário dos avanços tecnológicos contemporâneos, bem como diante do contexto de distanciamento social, como visto na pandemia da COVID-19, reduzindo alguns impactos educacionais e promovendo a aprendizagem ativa e centrada no aluno^2^,^3^,^5^. Contudo, é importante ressaltar que a dimensão temporal deste estudo compreende artigos publicados, em sua maioria, no período anterior à pandemia, onde não havia o que ficou conhecido como ensino remoto - no sentido emergencial de sua denominação. Faz-se necessário ponderar este fator porque os efeitos mentais provocados pelas medidas de enfrentamento da pandemia como o distanciamento social, isolamento domiciliar e outros em alunos e professores, além de impactos financeiros e aumento de vulnerabilidades sociais, têm sido apontados como interferências sobre o método do ensino híbrido^2^,^3^.

A presente revisão apontou que o ensino híbrido foi desenvolvido em diversos contextos de formação dos cursos da área da saúde, onde as TDICs foram apontadas como promissoras no desenvolvimento do ensino em saúde quando aliadas ao ensino presencial^16^,^18^,^21^,^22^,^47^. Também foi destacado que o uso do método misto no ensino das ciências da saúde poderá ser mais eficaz se forem capazes de remover os principais problemas do ensino exclusivamente tradicional, superando os problemas pertinentes ao ensino *online*^49^.

Estas evidências corroboram com os achados de uma metanálise sobre a efetividade do ensino híbrido para o aprendizado de profissionais de saúde, onde foi evidenciado que a possibilidade de realizar o autogerenciamento através da priorização dos conteúdos, definição do ritmo e local de estudo ou na contínua revisão dos materiais eletrônicos, pode ser uma explicação viável para o sucesso do ensino híbrido na aquisição do conhecimento^61^.

As amostras dos estudos analisados foram compostas por profissionais de diversas áreas da saúde, sejam eles enfermeiros, médicos, fisioterapeutas e outros, sugerindo que o ensino híbrido pode ser aplicado no contexto do ensino em saúde. Um estudo randomizado controlado recente, que investigou se a abordagem do ensino híbrido poderia melhorar os resultados de aprendizagem de profissionais de saúde em competências interprofissionais, concluiu que as estratégias de aprendizagem híbridas e tecnologicamente aprimoradas têm um impacto positivo no atendimento interprofissional em saúde e na satisfação dos alunos em relação ao aprendizado^62^.

Identificou-se que as atividades presenciais, com a realização de oficinas e *workshops*, precedidas de atividades *online* em ambientes virtuais contendo principalmente vídeos instrucionais e palestras interativas com especialistas foi a principal associação adotada nos programas de ensino híbrido analisados. Esta combinação, característica do modelo de sala de aula invertida, aumenta significativamente o nível de satisfação dos alunos e melhora o desempenho acadêmico em comparação com o ensino tradicional, pois aprimora a experiência educacional e os resultados somativos através de fontes de aprendizagem versáteis^63^,^64^.

Um dos estudos destacou que a aplicação da sala de aula invertida resultou em melhor desempenho dos alunos quando comparado com o ensino convencional, tendo como ponto positivo a aprendizagem colaborativa, a autonomia e a flexibilidade^18^. Em outras literaturas, foi visto que esta abordagem tem um grande potencial para desenvolver competências como o raciocínio clínico, pensamento crítico, comportamento comunicativo e capacidade de trabalho em equipe, sendo aplicável a educação de profissionais de saúde^64^,^65^.

Nesta mesma percepção, um estudo que investigou a eficácia da sala de aula invertida no desempenho acadêmico de alunos do curso de enfermagem ressaltou que houve melhor compreensão da aula presencial após leitura prévia do assunto em casa. Desta forma, percebese que o modelo de sala de aula invertida pode ser uma importante abordagem a ser utilizada na reformulação dos planejamentos de ensino na atualidade^6^,^64^,^65^.

Em um dos estudos analisados, uma simulação baseada na sala de aula invertida foi avaliada positivamente por estudantes de medicina, sendo ressaltado que esse modelo curricular, na percepção dos alunos, poderia ser aplicado também em outros estágios devido à utilização de vídeos educacionais sucintos, material de alto rendimento, temas práticos e relevantes e ao uso de múltiplas modalidades para reforçar o conhecimento, embora tenha havido pouca interação existente entre alunos e professores^52^. Percepção semelhante é encontrada na litaratura, visto que no modelo de sala de aula invertida os alunos enfrentam uma nova realidade e percebem que sua participação ativa é um fator direto de sucesso no que diz respeito à sua aprendizagem^64^,^65^. Ressalta-se que as estratégias educacionais devem ser interativas e diversificadas, como observado nos estudos revisados^11^,^32^,^55^,^59^. Comparadamente a isto, um estudo holandês que avaliou um programa para treinadores de médicos clínicos gerais mostrou que um programa multifacetado, incluindo diretrizes publicadas, estratégias adequadas, *feedback* oportuno e interação social pode resultarem melhorias no aprendizado^66^.

Apesar dos benefícios identificados, percebeu-se que existem desafios na autorregulação do estudante, pois nem todos os alunos são capazes de assumir o controle de seus estudos e regular com sucesso sua aprendizagem^19^. Foi apontado que isso decorre do fato dos alunos serem direcionados, desde a formação inicial, a aulas expositivo dialogadas, com o professor como centro do processo de ensino, o que os condiciona a almejarem que este método seja repetido para que se sintam seguros com os novos aprendizados^2^,^4^,^6^.

Isto também pode estar relacionado ao fato de que o uso das novas tecnologias aplicadas à educação enfrenta o grande desafio de motivar o aluno a buscar o conhecimento, mesmo estando fora da sala de aula^62^. Semelhantemente, identificaram a existência de desafios com o uso da tecnologia e dificuldades relacionadas à incompatibilidade de plataformas, e sugeriram que, para lidar com essas dificuldades, é necessário o fornecimento de diretrizes claras sobre os objetivos educacionais e tecnologias necessárias para desenvolver o programa^4^,^62^,^63^,^67^.

Diante do exposto, percebeu-se que o ensino híbrido vem ganhando cada vez mais destaque no ensino em saúde^20^. Foi visto que, a partir dele, o aluno destaca-se em sua aprendizagem porque é o principal gerenciador deste processo, aprendendo por diversos instrumentos educativos a partir da condução do professor^36^,^60^,^61^,^63^,^66^,^67^. Assim, fica evidente a importância do seu desenvolvimento na formação superior em saúde, especialmente em cenários de transição para a era educacional tecnológica, o que ficou ainda mais evidente durante a pandemia do SARS-COV-2.

## Conclusões

Por meio deste estudo, foi possível ilustrar como programas de ensino híbrido foram desenvolvidos e implementados ao redor do mundo, elucidando as principais contribuições deste método no processo de formação em saúde, além das estratégias e materiais de ensino mais utilizados e os desafios inerentes a este modelo de ensino. Os resultados apontam que o êxito do ensino híbrido pode estar relacionado ao seu caráter inovador, flexível, com uma boa relação custo-benefício e capaz de tornar os alunos protagonistas do seu processo de ensino aprendizagem, influenciando no desempenho acadêmico dos alunos.

O estudo apresentou limitações metodológicas quanto à impossibilidade de revisão dos arquivos sem acesso aberto, bem como ao acesso restrito de artigos pelo domínio da instituição de ensino ao qual esta pesquisa está vinculada. Outras limitações foram a não inclusão, no momento da construção do protocolo de revisão sistemática, bases de dados consideráveis como a *Red de Revistas Científicas de América Latina y el Caribe, España y Portugal* (Redalyc), *Web of Science* e *Cumulated Index to Nursing and Allied Health Literature*/*Elton B. Stephens Company* (CINAHL/Ebscho), o que poderia trazer ao estudo outras relevantes evidências.

Também é pertinente lembrar que a concentração dos estudos nos níveis de evidência baixos a moderados aponta para a necessidade de mais pesquisas que elucidem os potenciais efeitos do ensino híbrido na aprendizagem de estudantes da área da saúde, e assim direcionar a formulação de recomendações futuras para o seu desenvolvimento. Apesar disso, acredita-se que a compreensão das barreiras que envolvem a implementação deste modelo é fundamental para escolher aquele que melhor atenda às necessidades do público-alvo e às condições estruturais e tecnológicas das instituições de ensino. Conclui-se, então, que o ensino híbrido oferece intervenções capazes de aprimorar a formação superior em saúde.
